# SGLT-2 Inhibitor-Associated Euglycemic Diabetic Ketoacidosis: A Case Report and a Literature Review

**DOI:** 10.7759/cureus.26267

**Published:** 2022-06-23

**Authors:** Rabia Salman Mahfooz, Muhammad Khuzzaim Khan, Hussam Al Hennawi, Anwar Khedr

**Affiliations:** 1 Internal Medicine, Shalamar Institute of Health Sciences, Lahore, PAK; 2 College of Medicine, Dow University of Health Sciences, Civil Hospital Karachi, Karachi, PAK; 3 Internal Medicine, Jefferson Abington Hospital, Abington, USA; 4 Internal Medicine, BronxCare Health System, New York, USA

**Keywords:** : diabetes mellitus, diabetes mellitus type 2, empagliflozin, sodium-glucose cotransporter-2 (sglt-2) inhibitors, euglycemic diabetic ketoacidosis

## Abstract

Diabetic ketoacidosis (DKA) is considered a medical emergency, most commonly associated with type 1 diabetes mellitus, and is relatively rare in type 2 diabetes mellitus (T2DM). We discuss a case of a 45-year-old woman with T2DM who presented to the emergency room with worsening lethargy and weakness. Before her presentation, her physician had recently added empagliflozin, a sodium-glucose cotransporter-2 (SGLT-2) inhibitor, to her anti-diabetic drug regimen along with glimepiride and a combination drug of vildagliptin and metformin. Based on the clinical examination and lab findings, DKA was suspected, but her glucose level was below the cutoff value for DKA diagnosis. However, her lab results showed significant metabolic acidosis and ketonemia with no clinical or laboratory features of sepsis. Therefore, the diagnosis of euglycemic diabetic ketoacidosis (eu-DKA) was made. She was successfully treated according to the DKA protocol and discharged in good condition. In this report, our aim is to discuss the relationship between SGLT-2 inhibitors with eu-DKA. Given the absence of significant hyperglycemia, recognition of this entity by clinicians may be delayed. Serum ketones should be obtained in diabetic patients with symptoms of nausea, vomiting, or malaise while taking SGLT-2 inhibitors, and SGLT-2 inhibitors should be discontinued if ketoacidosis is confirmed.

## Introduction

Diabetic ketoacidosis (DKA) is diagnosed by the presence of a triad of hyperglycemia or random blood sugar (RBS) >250 mg/dL, metabolic acidosis (pH <7.3, serum bicarbonate <18 mEq/L), and ketosis [1]. Rarely does the patient present with characteristics of DKA in the presence of blood sugar levels less than 200 mg/dL, and this is defined as euglycemic diabetic ketoacidosis (eu-DKA) [2]. eu-DKA was first described by Munro et al. in 1973 [3]. This case documents one of the rare side effects associated with empagliflozin. Sodium-glucose cotransporter-2 (SGLT-2) inhibitors are a newer generation of anti-diabetic drugs that ultimately inhibit sodium-dependent glucose uptake by the kidneys and increase glucose loss in urine [4]. However, the diagnosis of DKA can sometimes be missed due to the presence of an associated euglycemic effect. Therefore, physicians should be aware when prescribing SGLT-2 inhibitors about this rare side effect and the novel presentation of eu-DKA

## Case presentation

We present here the case of a 45-year-old woman, a known case of type 2 diabetes mellitus (T2DM) for the last five years, poorly controlled with oral hypoglycemic medications including glimepiride, vildagliptin, and metformin. She saw her primary care physician, who added empagliflozin to the prescription five days prior to her presentation at the emergency department. She presented with complaints of increasing lethargy and weakness for the last two days. There were no preceding respiratory, gastrointestinal, or cardiovascular symptoms. She had no fever, cough, sore throat, chest pain, syncope, headache, vomiting, diarrhea, or urinary problems. She denied any history of chronic abdominal pain, weight loss, hypertension, ischemic heart disease, thyroid disease, stroke, substance abuse, or recent travel. She was married, a non-smoker, and a non-alcoholic. Her dietary habits were satisfactory. She had recently recovered from a COVID-19 infection two weeks ago.

On examination, she had no hyperthyroid or hypothyroid features. She had pallor and was slightly drowsy, but easily arousable; dehydrated but without jaundice and lymphadenopathy. Her pulse rate was 106 beats per minute (BPM) and regular, blood pressure (BP) was 110/70 mmHg with no postural drop, respiratory rate was 22 breaths per minute (BPM), peripheral capillary oxygen saturation (SpO_2_) was 88% at room air, and she was afebrile. Neurological examination showed normal tone, power, and reflexes, but she had bilaterally impaired vibration sense in her lower limbs. The rest of the systemic examination was unremarkable.

Her laboratory results revealed increased white blood cell (WBC) count and raised glycosylated hemoglobin (HbA1c) levels (Table 1). Analysis of arterial blood gas (ABG) and serum electrolytes revealed a decrease in pH, with hypobicarbonatemia, hypokalemia, and hypocarbia. A euglycemic state was indicated in basal metabolic tests with an elevated anion gap and ketosis. The patient also had no history of methanol, ethanol, and paraldehyde ingestion. Chest X-ray was unremarkable. Therefore, based on the clinical and laboratory findings, the diagnosis of eu-DKA due to the use of the SGLT-2 inhibitor was made.

**Table 1:** Baseline and post-recovery laboratory investigations WBC: white blood cell; MCV: mean corpuscular volume, AST: aspartate aminotransferases; ALT: alanine aminotransferase; RBS: random blood sugar.

Investigations	Day 1 - Before diagnosis	Day 4 - After recovery	Reference ranges
Complete blood count			
Hemoglobin	10.2 g/dL	10.1 g/dL	12-16 g/dL
WBC	15.7 × 10^3^ µL	11.4 × 10^3 ^µL	4-11 × 10^3^ µL
Platelets	215 × 10^3 ^µL	215 × 10^3 ^µL	150-400 × 10^3^ µL
MCV	91 fL	91 fL	80-100 fL
HbA1c	10.4%	10.4%	4.8-5.7%
Electrolytes			
Sodium	140 mEq/L	140 mEq/L	135-145 mEq/L
Potassium	2 mEq/L	3.2 mEq/L	3.5-5 mEq/L
Chloride	98 mEq/L	101 mEq/L	95-105 mEq/L
Arterial blood gas analysis			
pH	7.03	7.4	7.35-7.45
Bicarbonate	5.9 mmol/L	23.2 mmol/L	24-28 mmol/L
PCO_2_	19 mmHg	41 mmHg	35-45 mmHg
Basic metabolic panel			
RBS	142 mg/dL	133 mg/dL	<140 mg/dL
Ketones	>10 mmol/L	Negative	≤1.5 mmol/L
Lactate	13.8 mg/dL	13.2 mg/dL	4.5-19.8 mg/dL
Anion gap	36	15.4	8-16
Liver function tests			
AST	26 IU	29 IU	5-40 IU
ALT	29 IU	31 IU	5-40 IU
Renal function tests			
Creatinine	0.5 mg/dL	0.54 mg/dL	0.5-1.30 mg/dL
Urea	14 mg/dL	14 mg/dL	10-50 mg/dL
Urinalysis			
Urinary ketones	+++	Negative	<0.6

According to the local DKA protocol, the patient was treated with IV fluids, insulin, and potassium replacement. Four days later, the patient improved clinically. Her ABG, electrolyte levels, and basal metabolism returned to their normal values. After treatment, the patient was discharged in a stable condition.

## Discussion

The SGLT-2 inhibitors are a relatively new class of oral hypoglycemic agents that can be used as an adjunctive agent or alternative monotherapy for patients who fail initial therapy with lifestyle interventions, metformin, and/or sulfonylureas. Due to the obvious cardiovascular and renal benefits, they are preferred in patients with atherosclerotic cardiovascular disease, heart failure, or chronic kidney disease. These drugs dramatically reduce blood glucose and HbA1c level [5,6]. Additional benefits include promoting weight loss, decreasing blood pressure, reducing hypoglycemic episodes, and lowering exogenous insulin requirements [4]. However, several studies have estimated a three-fold increased risk of DKA compared to dipeptidyl peptidase-4 enzyme inhibitors [6]. We discuss the link between SGLT-2 inhibitors and eu-DKA.

Although SGLT-2 inhibitors are relatively safer drugs, they have been associated with side effects, such as urinary tract infection, genital mycotic infections, and volume depletion. eu-DKA has also been observed in rare cases [7]. Given the absence of significant hyperglycemia, the recognition of the problem may be delayed by both patients and clinicians. As noted in our case report, hyperglycemia was not present in our patient despite high anion gap metabolic acidosis and increased plasma and urinary ketones. Farjo et al. also studied a case of eu-DKA with empagliflozin in a 57-year-old man with T2DM with an RBS of 120 mg/dL after empagliflozin [8]. Hypoglycemia is associated with the use of SGLT-2 inhibitors, which in turn stimulate glucagon release and decrease glucose-dependent insulin release, thus altering the insulin-to-glucagon ratio and ketosis. Ketosis may be improved by reduced carbohydrate intake, starvation, acute illness, and decreasing exogenous insulin, as suggested by Burke et al. in their review [9]. Although SGLT-2 inhibitors increase glucose excretion in urine, this effect may be counterbalanced by its effect of increasing endogenous glucose production and glucagon release, which can eventually lead to ketosis [10,11]. In Pakistan, a case of eu-DKA after empagliflozin treatment was also reported [1]. Several similar cases have been reported in the literature. Some of them have been demonstrated in Table 2.

**Table 2:** Characteristics of similar reviewed cases DM: diabetes mellitus; T2DM: type 2 diabetes mellitus; HbA1c: glycosylated hemoglobin; WBC: white blood cells.

Study name	Age, years	Type of DM	Sex	SGLT-2 inhibitor	Blood analysis	Arterial blood gas	Basal metabolic panel	Renal function tests
Mistry and Eschler (2021) [12]	47	T2DM	Female	Empagliflozin	HbA1C: 13.6%	pH: 7.24. Serum bicarbonate: 11 mmol/L, β-hydroxybutyrate: 6.78 mmol/L	Plasma glucose: 187 mg/dL, anion gap: 22 mmol/L	Not reported
	34	T2DM	Male	Canagliflozin	HbA1C: 8.2%	pH: 7.27, serum bicarbonate: 12 mmol/L, β-hydroxybutyrate: 5 mmol/L	Serum glucose: 251 mg/dL, anion gap: 24 mmol/L	Not reported
Brown and McColl (2018) [13]	53	T2DM	Male	Dapagliflozin	WBC count: 12 × 10^3^ µL	pH: 7.24, β-hydroxybutyrate: 6.2 mmol/L	Blood glucose: 162 mg/dL, lactate: 4.5 mmol/L, anion gap: 30	Not reported
Chou et al. (2018) [14]	61	T2DM	Female	Dapagliflozin	Not reported	pH: 6.986, CO_2_: 20.9 mmHg, serum bicarbonate: 7.0 mEq/L	Blood glucose: 180 mg/dL, blood ketones: 8.0 mmol/L, urine ketones: positive, serum lactate: 9.0 mg/dL, anion gap: 20 mEq/L	Blood urea nitrogen: 25 mg/dL, serum creatinine: 0.8 mg/dL
Diaz-Ramos et al. (2019) [15]	44	T2DM	Female	Canagliflozin	Not reported	pH: 7.27, PCO_2_: 29 mm/Hg	Serum glucose: 163 mg/dL, serum bicarbonate: 14 mmol/L, anion gap: 18 mmol/L, urinary ketones: positive, serum acetone: positive	Not reported
Gajjar and Luthra (2019) [16]	28	T2DM	Female	Dapagliflozin	HbA1c: 10%	pH: 7.27, bicarbonate: 18 mmol/L, β-hydroxybutyrate: 5.29 mmol/L	Serum glucose: 111 mg/dL, anion gap: 20	Creatinine: 0.4 mg/dL
Lee and Ahn (2020) [17]	76	T2DM	Female	Dapagliflozin	WBC count: 11,800/μL, Hb: 13 g/dL, platelet count: 173,000/μL, erythrocyte sedimentation rate: 12 mm/hour, HbA1C: 8.1%	pH: 6.904, pCO_2_: 12.0 mmHg, serum bicarbonate: 3.1 mmol/L	Serum glucose: 410 mg/dL, insulin, 3.3 μIU/mL, anion gap; 37, serum lactate: 1.1 mmol/L, serum ketone: 2.7 mg/dL	Blood urea nitrogen: 41.7 mg/dL, creatinine: 3.2 mg/dL
Turner et al. (2016) [18]	62	T2DM	Female	Canagliflozin	HbA1c: 11.1 %	pH: 7.08, CO_2_: <5 mEq/L	Serum glucose: 213 mg/dL, anion gap: >17, serum lactate: 0.8 mmol/L	Blood urea nitrogen: 22 mg/dL, creatinine: 1.3 mg/dL
Steinmetz-Wood et al. (2020) [19]	47	T2DM	Male	Empagliflozin	HbA1c: 9.1%	pH: 6.94, serum bicarbonate: 5 mmol/L, β-hydroxybutyrate: 8.9 mmol/L	Serum glucose: 269 mg/dL, urinary ketones: +++, anion gap: 28	Creatinine: 1.21 mg/dL

**Figure 1:**
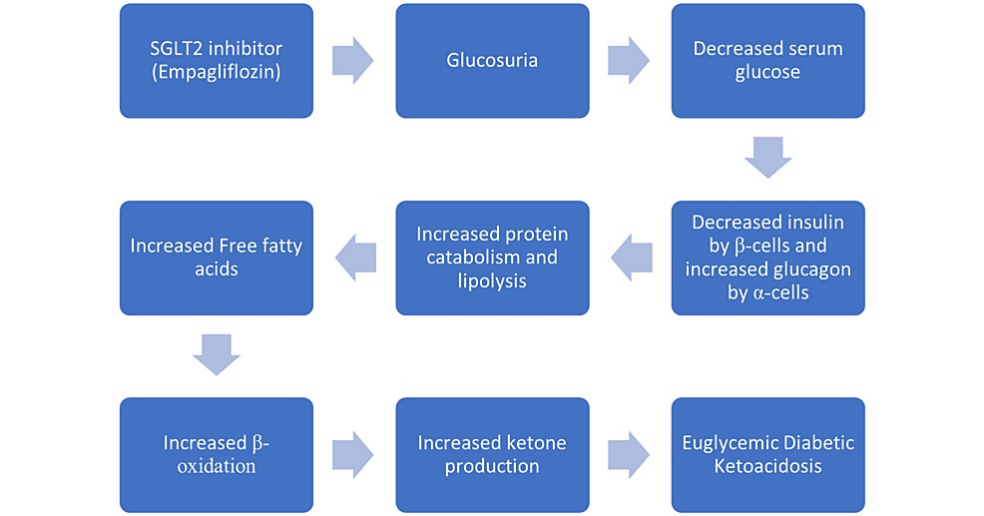
Mechanism of eu-DKA with SGLT-2 inhibitors. eu-DKA: euglycemic diabetic ketoacidosis; SGLT-2: sodium-glucose cotransporter-2.

This rare and unique form of DKA may be due to an altered physiological process. Usually, a decrease in insulin or insulin resistance causes a surge of glucagon release, leading to increased glucose levels and lipolysis, i.e., fatty acid oxidation and, hence, ketosis (Figure 1). A recent meta-analysis was able to develop a dose-dependent association between eu-DKA and SGLT-2 inhibitors. Patients taking high doses of SGLT-2 inhibitors have an increased risk of developing eu-DKA [20].

## Conclusions

The case report discusses a rare and life-threatening side effect of SGLT-2 inhibitors. Given the potential adverse consequences of eu-DKA, it is important that emergency physicians should consider the reasonable suspicion of eu-DKA in patients on SGLT-2 inhibitors, especially if the patient presents with nausea, vomiting, lethargy, dyspnea, and severe dehydration. Ketone studies and blood gas analyses would be performed on these patients, regardless of their blood glucose levels for early diagnosis and better prognostic outcomes. Patients taking SGLT-2 inhibitors should be educated to perform urine dipstick tests to check for ketonuria and seek immediate medical attention if positive.
